# Cerebral tuberculoma: an entity not to ignore

**DOI:** 10.11604/pamj.2016.24.133.9484

**Published:** 2016-06-10

**Authors:** Samia Frioui, Sonia Jemni

**Affiliations:** 1 Physical Medicine and Rehabilitation Department, Sahloul hospital, Sousse, Tunisia

**Keywords:** Cerebral tuberculoma, hemiplegia, CT scan

## Image in medicine

Cerebral tuberculomas are a rare and serious form of tuberculosis due to the haematogenous spread of Mycobacterium Tuberculosis. Symptoms and radiologic features are nonspecific, leading sometimes to misdiagnosis. We report the case of a 60-year-old male, with a history of diffuse bilateral infiltrative pulmonary disease at the stage of fibrosis, he made two generalized seizures associated with occipital headaches. CT scan showed a left frontal tumor, calcified lesion with edema around it. The patient was put under Depakine and corticosteroids. The evolution is marked by the occurrence of new seizures associated with heaviness of the right arm. Brain MRI showed a left posterior peripheral frontal meningioma with intralesional bleeding and significant edema around it with mass effect on the ipsilateral lateral ventricle. The patient was operated and the tumor was removed. In postoperative there was a Broca's aphasia with right hemiplegia. Pathological anatomical examination of the surgical specimen found a cerebromeningeal Granuloma with caseous necrosis in its pseudo tumor presentation (tuberculoma). The thoraco abdominal scan did not show any other tuberculosis lesions. The patient started antituberculosis treatment with 4 drugs (HRZE) for 2 months, followed by maintenance therapy (HR). The evolution was marked by the persistence of a right hemiplegia with Broca's aphasia. The patient was brought out in a wheelchair with functional rehabilitation sessions.

**Figure 1:**
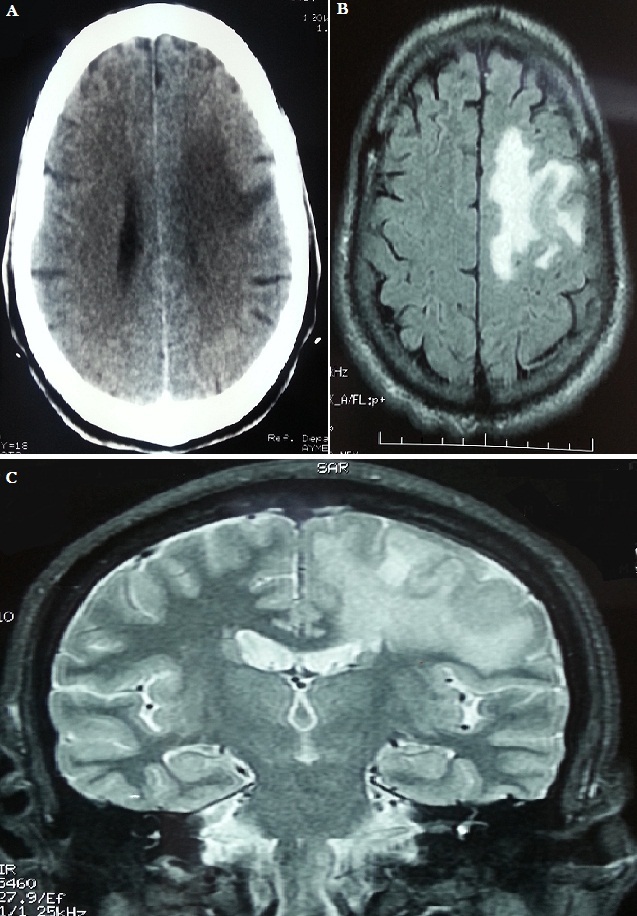
A) brain CT scan: a left frontal tumor process with peri-lesional oedema; B) axial brain MRI: a left posterior frontal peripheral meningioma with intralesional bleeding and significant perilesional oedema with mass effect on the ipsilateral lateral ventricle; C) frontal brain MRI section: a left posterior frontal peripheral meningioma with intralesional bleeding and significant peri-lesional oedema with mass effect on the ipsilateral lateral ventricle

